# Can Electrophysiological Parameters Substitute for Growth, and Photosynthetic Parameters to Characterize the Response of Mulberry and Paper Mulberry to Drought?

**DOI:** 10.3390/plants10091772

**Published:** 2021-08-25

**Authors:** Rui Yu, Yanyou Wu, Deke Xing

**Affiliations:** 1 Key Laboratory of Modern Agricultural Equipment and Technology, Ministry of Education, Institute of Agricultural Engineering, Jiangsu University, Zhenjiang 212013, China; 2111316005@stmail.ujs.edu.cn (R.Y.); xingdeke@ujs.edu.cn (D.X.); 2 Research Center for Environmental Bio-Science and Technology, State Key Laboratory of Environmental Geochemistry, Institute of Geochemistry, Chinese Academy of Sciences, Guiyang 550081, China

**Keywords:** *Morus alba* L., *Broussonetia papyrifera* (L.) Vent., physiological index, electrical signal, drought resistance

## Abstract

Drought is a key factor restricting plant survival, growth and development. The physiological parameters of plants are commonly used to determine the water status, in order to irrigate appropriately and save water. In this study, mulberry (*Morus alba* L.) and paper mulberry (*Broussonetia papyrifera* (L.) Vent.) seedlings were used as experimental materials, and four soil moisture treatments were set up for both plant species: 70–75% (CK: the control group, referred to as T0), 55–60% (T1: mild drought), 40–45% (T2: moderate drought), and 25–30% (T3: severe drought). The growth parameter of the plants was measured every two days from the onset of the treatment, the photosynthetic and electrophysiological parameters of the plants were measured every other week for a total of five times. The physiological responses and electrophysiological traits of leaves under different treatment levels were analyzed. The results showed that the photosynthetic and electrophysiological parameters could characterize the response of mulberry growth and development to soil water, and the growth and electrophysiological parameters could characterize the response of paper mulberry growth and development to soil water. Mild drought had no significant effects on the growth and development of mulberry and paper mulberry.

## 1. Introduction

Drought inhibits plant growth, thereby affecting agricultural production, and its global severity is great concern [1]. With the continuous intensification of the greenhouse effect, the frequency of drought is increasing [2]. Moreover, drought causes morphological and physiological changes, and even causes death [3]. Many parts of China, a large agricultural country, are currently suffering from drought, which not only restricts the development of agriculture and forestry, but also has adverse effects on the ecological environment [4]. Therefore, research on the drought resistance of plants has become a top priority.

Plants can respond differently to drought through complex regulatory mechanisms, which can be summarized as follows: (1) They can enhance water harvesting to escape drought, such as by developing roots, closing stomata, or lowering leaf temperature [5]. (2) They can respond to drought by reducing transpiration, through processes such as premature leaf shedding or accelerated leaf senescence [6]. (3) They can move prematurely from the vegetative to reproductive stage to speed up maturation and seed production before drought induces mortality [7]. (4) They can enhance their drought resistance by improving osmotic regulation and leaf tissue elasticity [8]. (5) The capacity for antioxidant metabolism can be increased to maintain normal growth under severe drought [9] and (6) Genetic mutation and genetic evolution of physiological and biochemical characteristics more adapted to drought can occur in long-term drought environments [10].

Some studies have found that morphological, physiological, molecular and other parameters can characterize the drought resistance of plants. The roots of the plant serve as sensing organs, and the leaves serve as expression organs, and together they coordinate internal or external defense mechanisms to resist drought [11,12,13,14]. To date, photosynthesis and water use efficiency have been widely used in the detection of drought tolerance in plants [15]. From the perspective of leaf morphology, leaf water loss and curling are important indicators of plant water status and drought resistance ability, which are typical responses of plants to water deficit [16]. Under drought conditions, stomatal conductance is the dominant factor restricting CO_2_ entry into cells, and stomatal development is greatly affected by environmental factors [17]. The accumulation of photosynthetic products in leaves is the basis of plant growth and yield. Stabilizing the photosynthetic production efficiency of crop populations is the goal of crop responses to drought [18].

Many indicators represent the ability of plants to resist drought. The morphological indexes of plants mainly describe the root system and leaf morphology. Leaves are the most sensitive organs of plants, and a change in leaf shape, such as the degree of wilting, can indicate the adaptability of plants to drought [19]. Physiological indexes including photosynthesis can also represent the drought resistance of plants, are widely used in the detection of drought tolerance in plants and can directly reflect the degree of water deficit [20]. Different levels of drought inhibit photosynthesis, and plants resist drought by closing their stomata, in turn reducing transpiration and CO_2_ loss to the external environment [21]. Cells, tissues and organs will be destroyed under drought conditions, which directly affects the accumulation of dry matter and is directly reflected in the yield [22]. Therefore, generally, old leaves of plants will fall off to resist drought. At the molecular level, drought resistance can be enhanced through the expression of specific genes, such as genes that promote the production of abscisic acid [23,24]. The traditional method for diagnosing drought resistance of plants is mainly based on the changes of plant morphological and physiological indexes [25]. Although the above indicators show that plants have their own advantages in terms of drought resistance, plant morphology must change gradually and remain static for a long time to reflect drought resistance [26]. Similar to photosynthetic indexes, morphological indexes cannot completely represent water use. Growth indicators need to be monitored for a long time, and sometimes they will be affected by light, leading to errors in the measured data [27,28]. Monitoring by molecular biology methods is relatively cumbersome and destructive. However, electrophysiological indexes can be used to monitor water use rapidly, online, accurately, in a timely manner, accumulatively, and nondestructively. Plants are organisms that adjust themselves to complex and changeable environments in the face of various stresses [29]. Plant electrophysiological signals are the most rapid and effective conduction signals between plant organs and tissues [30]. Most higher plants rely on plant electrophysiological signals to regulate their physiological functions [31]. Studies in recent years have shown that plant electrophysiological signal transducers are ubiquitous in higher plants and are the first response of plants to various stimuli in the external environment, which is reflected in plant growth, material metabolism and other aspects [32]. However, plant drought resistance can be analyzed by the initial response of photosynthesis, and electrophysiology has a strong correlation with this, so it can quickly reflect the photosynthetic characteristics of plants. Therefore, the study on changes of electrophysiological parameters is the basis for the realization of rapid detection of plant drought resistance.

Plant electrophysiological signals can quickly represent the internal growth conditions of plants and changes in the external environment, and thus have important physiological significance [31]. The capacitance (*C*_p_), leaf resistance (R), leaf impedance (Z) are the most common electrical parameters to evaluate the varying physiological status of plants [33]. When plant leaves are stimulated by the external environment, the cell membrane permeability changes immediately. Therefore, the electrolyte concentration in leaf cells inside and outside changes, leading to changes in *C*_p_, R, and Z in the leaves, and the water inside the cells also changes. The passive electrical properties of plant leaves (*C*_p_, R, and Z) can reflect plant water metabolism [34]. Many scholars have explored the response of plant electrophysiology through potential signals, resulting in vague biological significance. Studies of plant electrophysiology were most common until the late 1950s [35,36,37]. Electrical, hydrodynamic, and chemical signal transduction parameters also appear to be regulated when mimosa leaves are subjected to injury stress [38]. The transmission of electrical signals has an effect on photosynthesis and elicits a specific response in leaf photosynthesis [39,40,41]. Studies have shown a significant correlation between the impedance value of leaves and the relative water content, which can be used to accurately evaluate the water content of plants [42].

In recent years, plant electrical signals have been widely used in the field of plant stress resistance physiology in China. Li and Mao used impedance and capacitance to monitor the moisture content of tomato leaves in real time [43]. This research group has also successfully used electrical indicators to detect water status. Zhang et al. defined leaf tension and developed a model of the relationships between leaf tension, tissue water potential and physiological capacitance, which could be successfully applied to rapidly acquire water requirement information in *Brassica napus* during drought [44]. Xing et al. used the relationship between leaf tension and dry weight biomass to rapidly predict the rehydration time points of leaves under different drought treatments through online monitoring of electrophysiological parameters [45]. We also established a coupling model of leaf clamping force and physiological impedance and found that these parameters play an important role in photosynthesis and water use efficiency. In addition, the use of physiological impedance to monitor water status in plants is nondestructive and can determine the water demand of plants in a timely manner [46]. Since plant cell volume is closely related to physiological capacitance, impedance, resistance and capacitive resistance, electrophysiological characteristics are increasingly used in the diagnosis of plant water status [29,44]. Recently, many cutting-edge technologies have been applied to plant electrophysiology, and an increasing number of studies have been conducted on gene expression related to electrical signals [47].

Mulberry (*Morus alba* L., *M. alba*) and paper mulberry (*Broussonetia papyrifera* (L.) Vent., *B. papyrifera*), perennial tree species belonging to Moraceae, are characterized by a higher growth rate and greater adaptability to adverse environments than other species in this family [48]. They are widely valuable natural resources. The species *M. alba*., which is native to China and is now cultivated throughout the world [49], is also an economically important perennial tree, as it serves as the sole food source of the domesticated silkworm. In addition, it adds value through the production of edible fruit, timber and several pharmaceutically important chemicals [50]. *B. papyrifera*, which is a fast-growing tree mainly distributed in Asian and Pacific countries, can be utilized in many applications. It is well known for its bark fibers, which are used to manufacture high-quality paper, cloth, ropes, animal feed, bioenergy feedstock and traditional Chinese medicines [51,52]. Based on previous studies [53], the two plants have great differences in drought resistance and different mechanisms of water use efficiency. This paper can be used as a comparison material to further study the different characteristics of their electrophysiological responses to drought.

The main contents of this paper are as follows: First, we study the responses of the electrophysiological, photosynthetic and growth parameters of the two plant species to different water treatments. Second, we study the relationships between the electrophysiological, photosynthetic and growth parameters. Finally, we study the different mechanisms underlying the response to drought between the two plant species. Based on these studies, this paper mainly aims to explore the feasibility of using electrophysiological parameters to characterize plant water metabolism, and to clarify the response mechanism of two plant species to drought by combining electrophysiological, photosynthetic and growth parameters.

## 2. Results

### 2.1. Soil Moisture Content under Different Drought Levels

As illustrated in Table 1, the moisture content of the soil in which *M. alba* and *B.* *papyrifera* were grown varied depending on the stress level. At the level of T0, the soil moisture content of *M. alba* and *B. papyrifera* was the maximum, and it decreased gradually in subsequent levels. At the level of T3, the soil moisture content of *M. alba* and *B. papyrifera* was the minimum.

### 2.2. Equation-Derived Growth Differences of M. alba and B. papyrifera under Different Water Stress Levels

As illustrated in Table 2 and Table 3, the 4-parameter logistic equation fit the plant height, leaf length and leaf width data of *M. alba* and *B. papyrifera* under the different water stress levels well. As shown in Table A1 and Table A2 in the Appendix A, the coefficient of determination (R^2^) of all fitted equations is greater than 0.99, and *p* < 0.0001, indicating that the 4-parameter logistic equation can characterize the relationships between plant height, leaf length and leaf width over time. Based on the GR_50_ and DT_log_ values estimated by the 4-parameter logistic equation, the plant height, leaf length, leaf width, and leaf area growth of *M. alba* and *B. papyrifera* under drought conditions were calculated (Table 4 and Table 5).

As shown in Table 4, the plant height of *B. papyrifera* at the T0 level was significantly higher than that at other levels, but the differences were not significant. The leaf length of *B. papyrifera* at the T0 and T1 levels was significantly greater than that at the T2 and T3 levels, and it was minimal at the T3 level. The leaf width of *B. papyrifera* at the T0 and T1 levels was significantly higher than that at the T2 and T3 levels. Therefore, the leaf area of *B. papyrifera* at the T0 and T1 levels was significantly greater than that at the T2 and T3 level, that at the T0 level was the maximal, and that the T3 level was the minimal. Compared with that in T0, the GR_50_ × DT _log_ of *B. papyrifera* in T1 decreased by 7.68%, T2 decreased by 51.57% and T3 decreased by 59.67%. However, as shown in Table 5, the plant height, leaf length, leaf width and leaf area of *M. alba* were not significantly different among the water stress treatments.

### 2.3. Photosynthetic Parameters of M. alba and B. papyrifera under Different Water Stress Levels

#### 2.3.1. The Net Photosynthetic Rate (*P*_N_)

**Figure 1:**
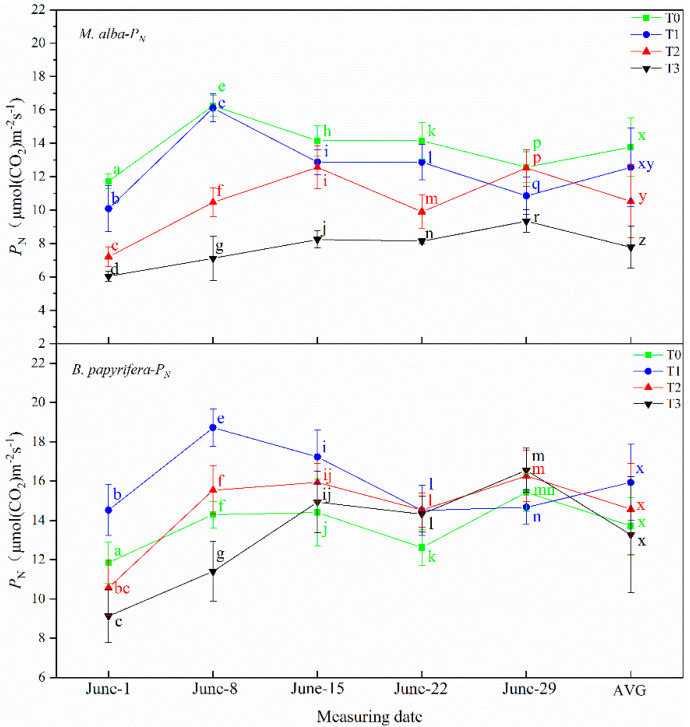
The net photosynthetic rate (*P*_N_) of the leaves of *M. alba* and *B. papyrifera* under different water stress levels. Note: Values are the means ± SE of 15 replicates. Different letters indicate significant differences at T0–T3 levels in each measurement. Bars with different letters are significantly different at *p* ≤ 0.05 (Tukey). AVG is the mean value of five measured data. *M. alba*-*P*_N_: The net photosynthetic rate of the leaves of *M. alba*. *B. papyrifera*-*P*_N_: The net photosynthetic rate of the leaves of *B. papyrifera*. T0 is the control group, T1 is the mild drought, T2 is the moderate drought, T3 is the severe drought.

The leaf net photosynthetic rate of *M. alba* and *B. papyrifera* at different treatment levels was measured five times every other week, measurements began on 1 June, after 7 d of drought stress for 8 June, 14 d of drought stress for 15 June, 21 d of drought stress for 22 June, 28 d of drought stress for 29 June, as illustrated in Figure 1.

The net photosynthetic rate of *M. alba* at the T0 level was significantly higher than that at other levels at all drought times, that at the T3 level was the lowest, and the AVG showed the same characteristics. On 1 June, 8 June and 15 June, the net photosynthetic rate of *B. papyrifera* was significantly higher at the T1 level than at the other levels, and that at the T3 level was minimal on 1 June and 8 June. No significant differences were observed on 22 June and 29 June. There was no significant difference in the AVG among the 4-levels.

#### 2.3.2. The Transpiration Rate (*Tr*)

**Figure 2:**
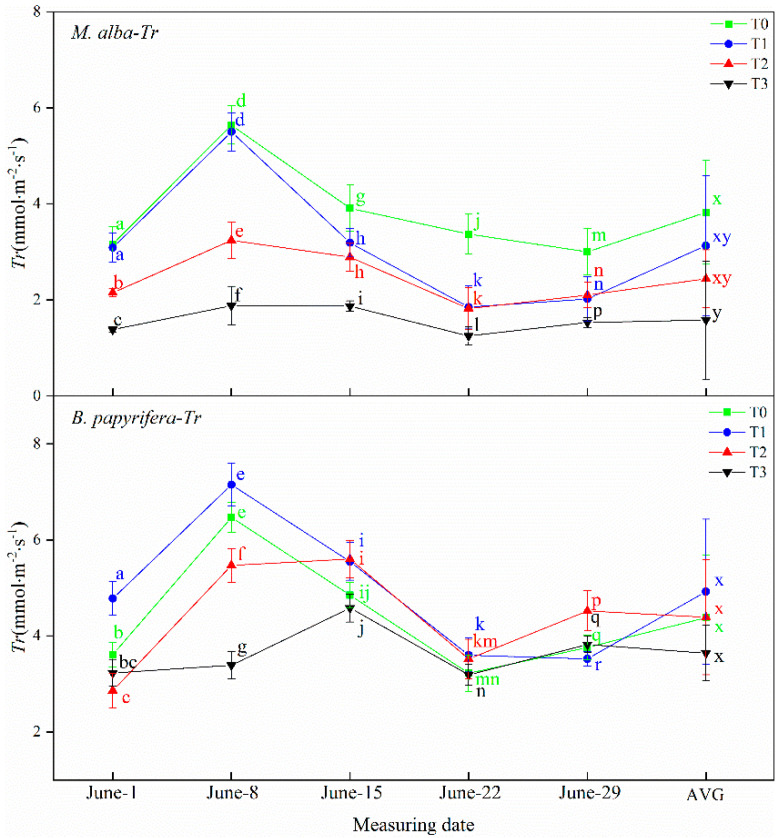
The transpiration rate (*T*r)of the leaves of *M. alba* and *B. papyrifera* under different water stress levels. Note: Values are the means ± SE of 15 replicates. Different letters indicate significant differences at T0-T3 levels in each measurement. Bars with different letters are significantly different at *p* ≤ 0.05 (Tukey). AVG is the mean value of five measured data. *M. alba*-*T*r: The transpiration rate of the leaves of *M. alba*. *B. papyrifera*-*T*r: The transpiration rate of the leaves of *B. papyrifera*. T0 is the control group, T1 is the mild drought, T2 is the moderate drought, T3 is the severe drought.

The transpiration rate of *M. alba* and *B. papyrifera* showed differences among the water stress levels (Figure 2). The transpiration rates of *M. alba* showed obvious differences, with the highest value observed at the T0 level, progressively lower values observed at the T1 and T2 levels, and the lowest value observed at the T3 level, except on 29 June. This pattern was consistent with that observed for the AVG, where the transpiration rate of *M. alba* was also maximal at T0 and minimal at T3. However, on 1 June, 8 June and 22 June, the transpiration rate of *B. papyrifera* at the T1 level was significantly higher than that at the other levels. Moreover, the transpiration rate of *B. papyrifera* at the T3 level was the lowest on 8 June, 15 June and 22 June, but on 29 June, that at the T1 level was the lowest, and that at the T2 level was the highest.

#### 2.3.3. Stomatal Conductance (*g_s_*)

**Figure 3:**
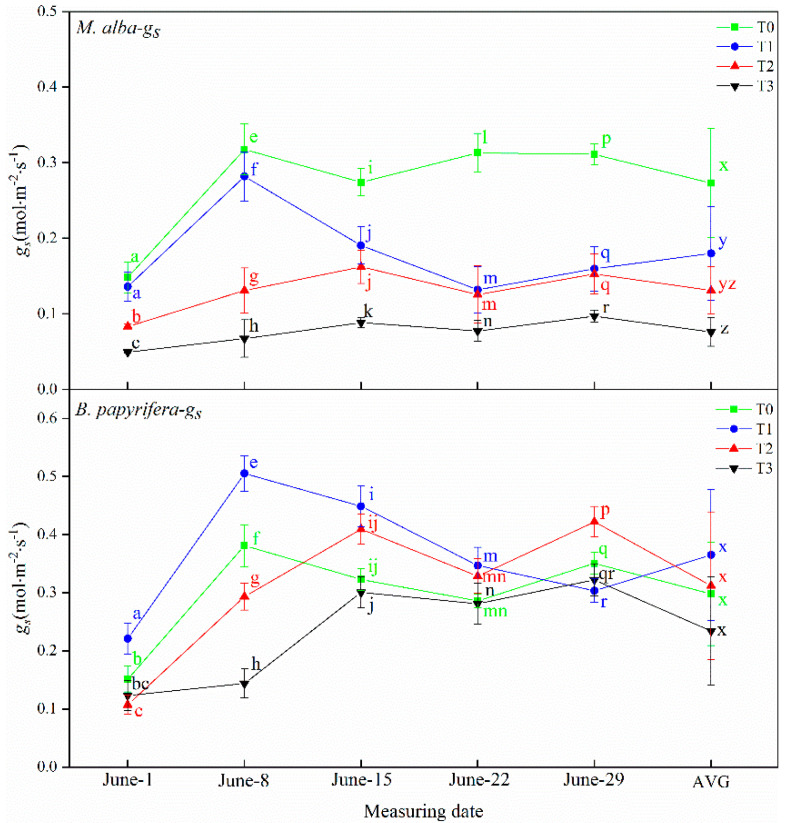
The stomatal conductance (*g_s_*)of the leaves of *M. alba* and *B. papyrifera* under different water stress levels. Note: Values are the means ± SE of 15 replicates. Different letters indicate significant differences at T0–T3 levels in each measurement. Bars with different letters are significantly different at *p* ≤ 0.05 (Tukey). AVG is the mean value of five measured data. *M. alba*-*g_s_*: The stomatal conductance of the leaves of *M. alba*. *B. papyrifera*-*g_s_*: The stomatal conductance of the leaves of *B. papyrifera*. T0 is the control group, T1 is the mild drought, T2 is the moderate drought, T3 is the severe drought.

The stomatal conductance of *M. alba* and *B. papyrifera* under different water stress levels is presented in Figure 3. The stomatal conductance of *M. alba* was highest at the T0 level, followed by the T1, T2, and then T3 level, thus showing obvious regularity. The same pattern was observed for the AVG. However, on 29 June, the stomatal conductance of *B. papyrifera* at the T1 level was higher than that at all other levels, on 8 June, 15 June and 22 June, that at the T3 level was the lowest. On 29 June, the stomatal conductance of *B. papyrifera* was lowest at the T1 level and highest at the T2 level.

#### 2.3.4. The Intercellular CO_2_ Concentration (*C*_i_)

**Figure 4:**
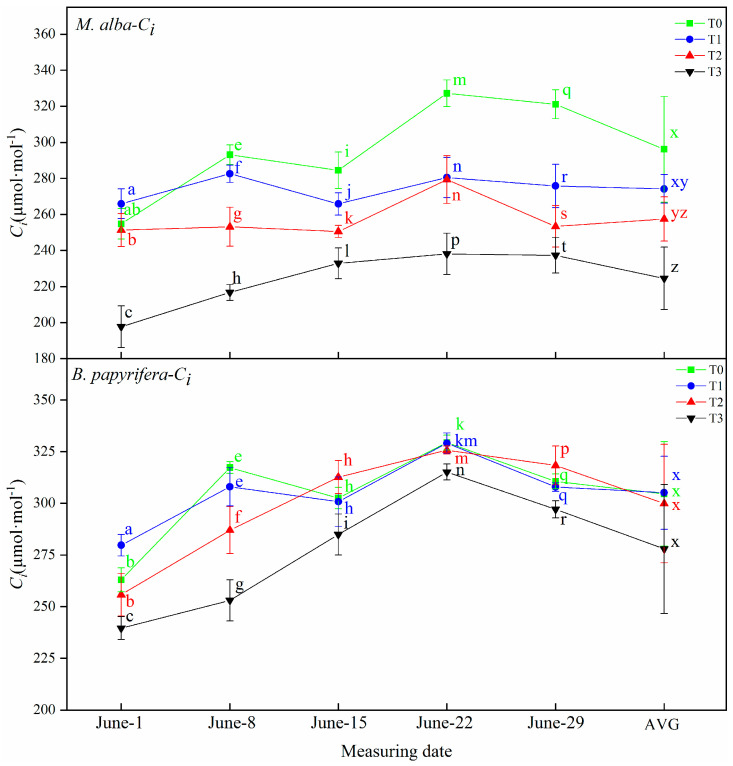
The intercellular CO_2_ concentration (*C_i_*) of the leaves of *M. alba* and *B. papyrifera* under different water stress levels. Note: Values are the means ± SE of 15 replicates. Different letters indicate significant differences at T0-T3 levels in each measurement. Bars with different letters are significantly different at *p* ≤ 0.05 (Tukey). AVG is the mean value of five measured data. *M. alba*-*C_i_*: The intercellular CO_2_ concentration of the leaves of *M. alba*. *B. papyrifera*-*C_i_*: The intercellular CO_2_ concentration of the leaves of *B. papyrifera*. T0 is the control group, T1 is the mild drought, T2 is the moderate drought, T3 is the severe drought.

The intercellular CO_2_ concentration of *M. alba* and *B. papyrifera* under different water stress levels is shown in Figure 4. The intercellular CO_2_ concentration of *B. papyrifera* showed obvious regularity. On 1 June, that at the T1 level was slightly higher than that at the T0 level, while at other treatment times, the concentration was highest at the T0 level and sequentially lower at the T1–T3 levels, with significant differences observed between the water stress levels. The AVG showed the same characteristics. However, on 1 June, the intercellular CO_2_ concentration of *B. papyrifera* at the T1 level was higher than that at the other levels, and that at the T3 level was the lowest. In terms of the intercellular CO_2_ concentration of *B. papyrifera*, the water stress levels were ordered as T0 > T1 > T2 > T3 on 8 June and 22 June, but on 15 June and 29 June, the value at T2 was the highest, and that at T3 was the lowest.

#### 2.3.5. Water Use Efficiency (WUE)

**Figure 5:**
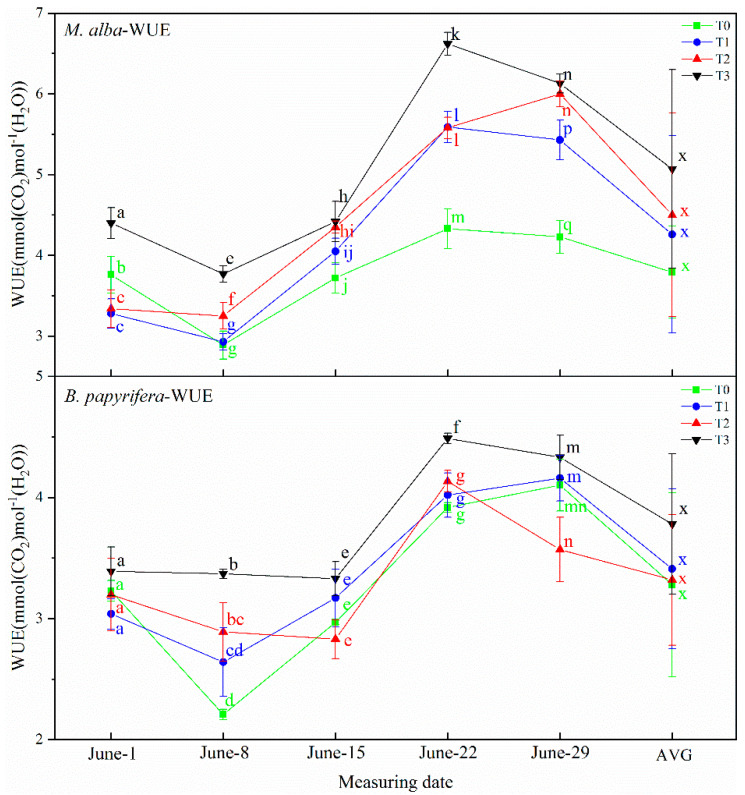
The water use efficiency (WUE)of the leaves of *M. alba* and *B. papyrifera* under different water stress levels. Note: Values are the means ± SE of 15 replicates. Different letters indicate significant differences at T0–T3 levels in each measurement. Bars with different letters are significantly different at *p* ≤ 0.05 (Tukey). AVG is the mean value of five measured data. *M. alba*-WUE: The water use efficiency of the leaves of *M. alba*. *B. papyrifera*-WUE: The water use efficiency of the leaves of *B. papyrifera*. T0 is the control group, T1 is the mild drought, T2 is the moderate drought, T3 is the severe drought.

The water use efficiency of *M. alba* and *B. papyrifera* under different water stress levels is shown in Figure 5. The water use efficiency of *M. alba* showed the same regularity on 8 June, 15 June and 29 June measurement, where it was highest at T0 and decreased sequentially from T1–T3. These differences were significant, and the AVG showed the same characteristics. On 22 June, the concentration at T1 was slightly higher than that at T2, but the difference was not significant. On 1 June, the water use efficiency of *M. alba* was highest at the T3 level and lowest at the T1 level, but there were no significant differences between the T0, T1 and T2 levels. The AVG showed the same characteristics. At each treatment time, the water use efficiency of *B. papyrifera* was highest at the T3 level. On 8 June and 22 June, the water use efficiency of *B. papyrifera* was lowest at T0, and highest at T3. On 1 June, it was lowest at the T3 level, and on 15 June and 29 June, it was lowest at T2 and highest at T3.

### 2.4. Electrophysiological Parameters of M. alba and B. papyrifera under Different Water Stress Levels

#### 2.4.1. Physiological Capacitance (*C*_p_)

**Figure 6:**
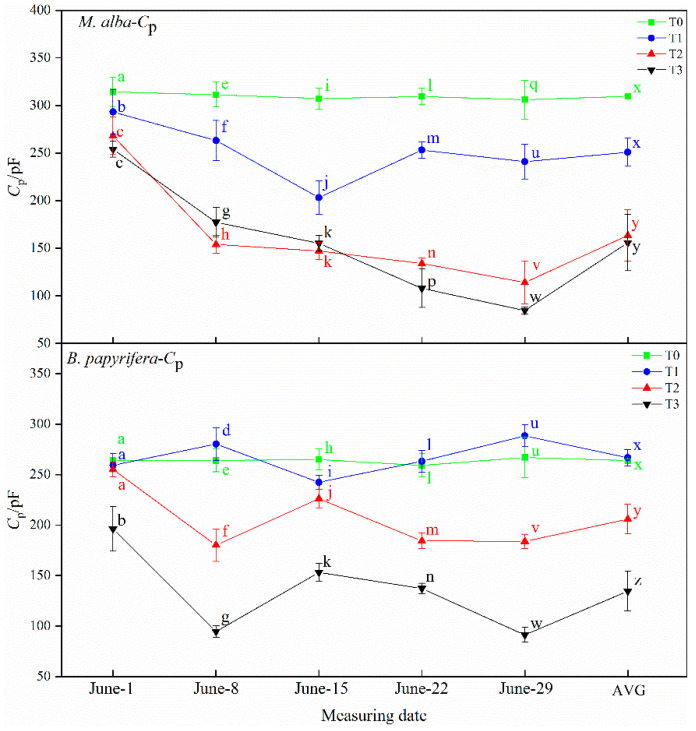
The physiological capacitance (*C*_p_) of the leaves of *M. alba* and *B. papyrifera* under different water stress levels. Note: Values are the means ± SE of 15 replicates. Different letters indicate significant differences at T0-T3 levels in each measurement. Bars with different letters are significantly different at *p* ≤ 0.05 (Tukey). AVG is the mean value of five measured data. *M. alba*-*C*_p_: The physiological capacitance of the leaves of *M. alba*. *B. papyrifera*-*C*_p_: The physiological capacitance of the leaves of *B. papyrifera*. T0 is the control group, T1 is the mild drought, T2 is the moderate drought, T3 is the severe drought.

As illustrated in Figure 6, the physiological capacitance of *M. alba* and *B. papyrifera* varied with water stress level. The physiological capacitance of *M. alba* at different drought times showed a decreasing trend across the four water stress levels. The AVG showed the same characteristics. On 8 June and 15 June, although the physiological capacitance of *M. alba* at the T3 level was higher than that at the T2 level, but the AVG showed the decreasing trend. The physiological capacitance of *B. papyrifera* at different drought times also showed a decreasing trend across the four water stress levels. On 8 June, 22 June and 29 June, although the physiological capacitance of *B. papyrifera* at the T1 level was higher than that at the T0 level, but the AVG showed the decreasing trend.

#### 2.4.2. Physiological Resistance (R)

**Figure 7:**
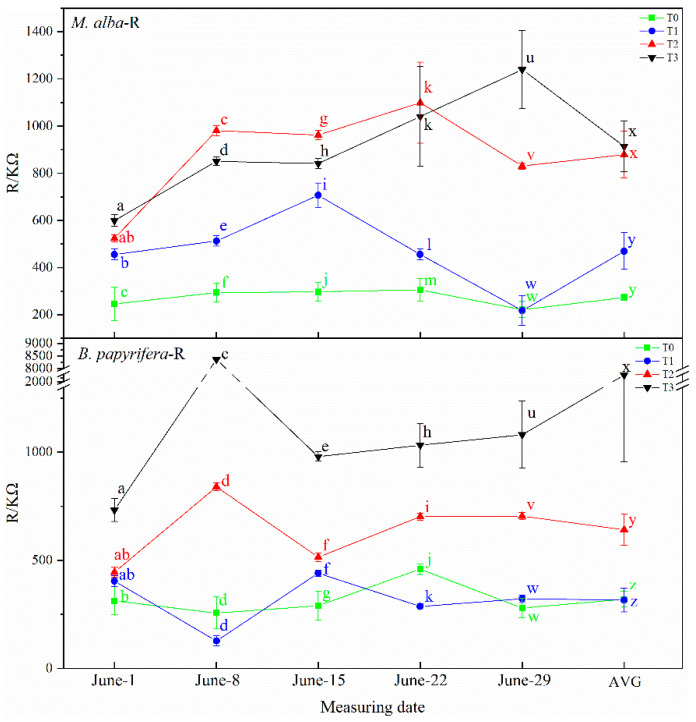
The physiological resistance (R) of the leaves of *M. alba* and *B. papyrifera* under different water stress levels. Note: Values are the means ± SE of 15 replicates. Different letters indicate significant differences at T0–T3 levels in each measurement. Bars with different letters are significantly different at *p* ≤ 0.05 (Tukey). AVG is the mean value of five measured data. *M. alba*-R: The physiological resistance of the leaves of *M. alba*. *B. papyrifera*-R: The physiological resistance of the leaves of *B. papyrifera*. T0 is the control group, T1 is the mild drought, T2 is the moderate drought, T3 is the severe drought.

As illustrated in Figure 7, the physiological resistance of *M. alba* and *B. papyrifera* varied with water stress level. The physiological resistance of *M. alba* at different drought times showed increasing trend across the four water stress levels. On 8 June, 15 June and 22 June, although the physiological resistance of *M. alba* at the T2 level was higher than that at the T3 level, but the AVG showed the increasing trend. The physiological resistance of *B. papyrifera* at different drought times showed an increasing trend across the four water stress levels. On 8 June and 22 June, although the physiological resistance of *B. papyrifera* at the T0 level was higher than that the T1 level, but the AVG showed the increasing trend.

#### 2.4.3. Physiological Impedance (Z)

**Figure 8:**
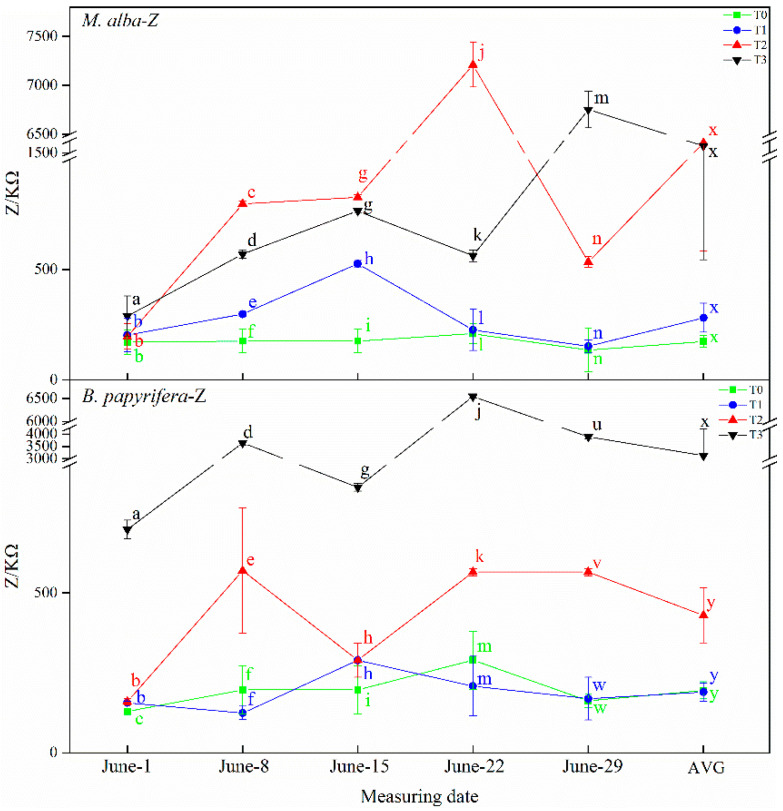
The physiological impedance (Z) of the leaves of *M. alba* and *B. papyrifera* under different water stress levels. Note: Values are the means ± SE of 15 replicates. Different letters indicate significant differences at T0–T3 levels in each measurement. Bars with different letters are significantly different at *p* ≤ 0.05 (Tukey). AVG is the mean value of five measured data. *M. alba*-Z: The physiological impedance of the leaves of *M. alba*. *B. papyrifera*-Z: The physiological impedance of the leaves of *B. papyrifera*. T0 is the control group, T1 is the mild drought, T2 is the moderate drought, T3 is the severe drought.

As illustrated in Figure 8, the physiological impedance of *M. alba* and *B. papyrifera* varied with water stress level. The physiological impedance of *M. alba* at different drought times showed an increasing trend across the four water stress levels. On 8 June and 15 June, although the physiological impedance of *M. alba* at the T2 level was higher than that at the T3 level, but the AVG showed the increasing trend, and the difference of T2 and T3 levels were not significant. However, on 22 June, the physiological impedance of *M. alba* at the T2 level was obviously higher than that at the other levels, but the AVG showed the increasing trend. In the same way, the physiological impedance of *B. papyrifera* at different drought times showed an increasing trend across the four water stress levels, with that observed at the T3 level significantly higher than that observed at the other levels. On 8 June, 22 June and 29 June, although the physiological impedance of *B. papyrifera* at the T0 level was higher than that at the T1 level, the difference was not significant.

### 2.5. Relationship between Different Physiological Parameters of M. alba and B. papyrifera

The Pearson correlation coefficients for the relationships between the different physiological properties of *M. alba* and *B. papyrifera* are shown in Table 6 and Table 7. In *M. alba*, *P*_N_ was strongly positive correlated with *g_s_*, *C*_i_, *Tr* and *C*_p_ (0.888 **, 0.685 **, 0.850 ** and 0.380 ** respectively), negatively correlated with WUE (−0.319 *), and highly negative correlated with R (−0.425 **) (Table 6). *g_s_* was found to be strongly positively correlated with *C*_i_, *Tr* and *C*_p_ (0.834 **, 0.820 ** and 0.546 **, respectively) and strongly negatively correlated with WUE and R (−0.413 ** and −0.532 **, respectively). *C*_i_ was strongly positively correlated with *Tr* and *C*_p_ (0.573 ** and 0.480 **, respectively), negatively correlated with WUE (−0.280 *), and strongly negatively correlated with R (−0.482 **). *Tr* was found to be strongly negatively correlated with WUE and R (−0.709 ** and −0.464 **, respectively), strongly positively correlated with *C*_p_ (0.544 **), and negatively correlated with Z (−0.311 *). WUE was strongly negatively correlated with *C*_p_ (−0.585 **) and strongly positively correlated with R and Z (0.395 ** and 0.410 **, respectively). *C*_p_ was strongly negatively correlated with R and Z (−0.892 ** and −0.545 **, respectively). R was strongly positively correlated with Z (0.600 **).

In *B. papyrifera*, *P*_N_ was strongly positively correlated with *g_s_*, *C*_i_ and *Tr* (0.861 **, 0.535 ** and 0.719 **, respectively). *g_s_* was strongly positively correlated with *C*_i_ and *Tr* (0.725 ** and 0.753 **, respectively), and *g_s_* was negatively correlated with R (−0.289 *). *C*_i_ was strongly positively correlated with *Tr* (0.356 **) and strongly negatively correlated with R (−0.369 **). *Tr* was strongly negatively correlated with WUE (−0.684 **) and negatively correlated with Z (−0.294 *). WUE was negatively correlated with *C*_p_ (−0.281 *), and strongly positively correlated with Z (0.445 **). *C*_p_ was strongly negatively correlated with R and Z (−0.548 ** and −0.712 **, respectively). R was strongly positively correlated with Z (0.471 **).

## 3. Discussion

Almost all life activities in plants involve water. Plant physiological information can be represented by the leaf physical parameters which can be directly measured and ensure the accuracy and improve the convenience of drought resistance research of plants [54]. The determination of electrophysiological indexes provides a more convenient method for the study of water status in cells. Electrical signals of plants are considered to be the fastest response of plants to environmental changes and are closely related to the life activities of plants such as material and energy metabolism, development, stress resistance and signal transduction [27,30]. When plant cells lose water and contract, resulting in smaller cell size, the cell expansion pressure must change. Capacitance is related to the degree of expansion and contraction of plant cells [55]. At the same time, the change of water content will inevitably lead to the change of membrane permeability and ion concentration inside and outside the cell, which is closely related to the resistance and impedance [56]. When plants are subjected to different degrees of environmental stress, the water status, ion concentration and membrane permeability of cells will change immediately, and the electrical signals of plants will also change. Therefore, the electrophysiological indexes have the theoretical basis to reflect the water status of plant leaves [57,58]. In this paper, the response of *M. alba* to drought can be characterized by photosynthetic parameters rather than growth parameters. Drought can cause a decrease in plant cell turgor pressure, which leads to a decrease in components of the plant growth rate, such as plant height, stem thickness and leaf area [59]. Drought has a strong impact on plant growth and development and causes death in severe cases. In addition, the response of *B. papyrifera* to drought can be characterized by growth parameters rather than photosynthetic parameters. Drought will affect plant photosynthesis, leaf photosynthesis is an important physiological activity of plants, and the accumulated products of photosynthesis play an important role in plant growth and development [60]. However, electrophysiological parameters can characterize the response of *M. alba* and *B. papyrifera* to drought. The degree of drought in plants is related to the electrophysiological characteristics of leaves. Therefore, electrophysiological parameters can be used to quickly and nondestructively determine the water status of leaves.

To characterize the effect of drought, the growth curves were analyzed initially using the logistic model, the adequacy of which is discussed. Under different water conditions, the growth models of different plant species are also different [61]. The sigmoidal model measures the growth of plants over time and thus can be used to analyze the temporal growth trend of plants [62]. In this study, a 4-parameter logistic model was used to fit the growth parameters of *M. alba* and *B. papyrifera*, such as X_0_, a, GR_50_, DT_log_, DT_s_, and R^2^, under different drought levels, and the physiological parameters plant height, leaf length, leaf width and leaf area were analyzed. The results obtained by the 4-parameter logistic model in this study revealed obvious patterns in the growth parameters of *B. papyrifera* under different drought levels. Changes in the plant height, leaf length, leaf width and leaf area parameters of *B. papyrifera* showed obvious regularity with the decrease in soil moisture. These results reveal specific life phenomena in plants; that is, *B. papyrifera* grows fast and has a long-life cycle, and its growth is affected by drought at any treatment time. In contrast, the life cycle of *M. alba* is shorter, its photosynthesis is lower, and its overall growth is slower. Moreover, the logarithmic growth period of *M. alba* passed quickly, so drought had no effect on its leaf growth.

Drought resistance in plants can be determined through their photosynthetic and physiological characteristics. The main driving force for the growth of plants is photosynthesis, which supplies the energy and carbon required for the biosynthesis of organic compounds necessary for development [63]. Increasing photosynthetic energy use efficiency and enhanced photosynthetic capacity may be the most successful mechanisms for alien species invasion and for adaptability to adverse environments [64]. In response to water deficiency, stomatal closure occurs, which reduces the net photosynthetic rate and transpiration rate and decreases the intercellular CO_2_ concentration [65]. In this study, the leaf area of *B. papyrifera* is larger than *M. alba*, and the photosynthesis is stronger than *M. alba*, the photosynthetic parameters of *M. alba* responded to soil moisture at different time under different drought treatments, different from *M. alba*, the photosynthetic parameters of *B. papyrifera* only responded to soil water in the early stage, but did not respond to soil water in the later stage, indicating that *B. papyrifera* has strong adaptability. Although the area of *M. alba* leaves was small, the photosynthetic parameters exhibited obvious differences under different drought levels, indicating that *M. alba* has a more obvious response than *B. papyrifera* to water. *B. papyrifera* continues to grow fast and can be regarded as a “pool”. It needs to consume energy for growth. The carbohydrates produced via photosynthesis cannot be consumed through growth, thus inhibiting photosynthesis. Therefore, photosynthetic parameters cannot characterize the response of *B. papyrifera* to drought. *M. alba* leaves are small and grow slowly, so the pool is small, which affects photosynthesis. Photosynthesis does not affect the growth of individual leaves. The substance produced via photosynthesis are used to promote the growth of new leaves. *M. alba* leaves are abundant. Therefore, photosynthetic characters can characterize the response of *M. alba* to drought.

Plants differ in their physiological capacitance response to water stress. Burdon-Sanderson noted the electrical phenomena that accompany irritation of the leaf of *Dionaea muscipula* [66,67], prompting studies of plant electrical signals. Previous studies showed that direct changes in plant electrophysiological information such as Z, R and *C*_p_ could directly reflect changes in plant water [68,69]. Jamaludin et al. study showed that there was a significant correlation between the impedance value of leaves and relative water content, and the water content of plants could be accurately evaluated by measuring the impedance [42]. Yang et al. studied the relationship between plants and air heat in the greenhouse and found that the growth status of plants could be determined by measuring the changes of plant leaf temperature, transpiration and surface electrophysiology of plants [70]. By studying wheat, Briggs found that there was a logarithmic relationship between the water content of wheat and its physiological resistance, and the water content of wheat could be calculated indirectly by measuring the physiological resistance [71]. Ksenzhek et al. studied the physiological resistance of the main vein of maize in different directions, and the results showed that the physiological resistance of the leaf gradually decreased with the wilting of the leaf [72]. In addition, the water status data of plant leaves obtained by photosynthesis-transpiration and water potential did not directly reflect the intracellular water status [73,74]. Through the study of scholars, it is found that the monitoring and analysis of the changes of electrical signals and physiological and biochemical aspects of plants under drought stress, and the test of the response of electrophysiological characteristics of plants to drought stress, so as to achieve a comprehensive, timely and accurate characterization of the drought resistance of crops under drought stress. In this study, the leaf electrophysiological parameters (*C*_p_, R and Z) of *M. alba* and *B. papyrifera* accurately revealed the diversity of intracellular water metabolism in plant leaves and could potentially be applied in the acquisition of plant water information. The results of this study revealed obvious characteristics of the electrophysiological parameters of *M. alba* and *B. papyrifera* With a gradual increase in plant water shortage severity, the capacitance value of *M. alba* and *B. papyrifera* decreased gradually and the water content of leaves decreased gradually, and with the increase in resistance and impedance, stomata closed, transpiration decreased, leaf water use efficiency increased, and soil water decreased. Therefore, the electrophysiological index can respond to soil water shortages.

In this study, correlation analysis between photosynthetic and electrophysiological parameters of *M. alba* and *B. papyrifera* was performed for the first time (Table 6 and Table 7). Plant physiological resistance and intercellular CO_2_ concentration are strongly negatively correlated in the two species. The physiological resistance of *M. alba* and *B. papyrifera* is also strongly negatively correlated with stomatal conductance; the greater the physiological resistance is, the smaller the intercellular CO_2_ concentration. This explains why physiological resistance determines stomatal resistance and physiological resistance leads to greater stomatal resistance and smaller intercellular CO_2_ concentrations. The osmotic potential of leaf cells will change immediately upon exposure to drought, so the electrolyte concentration will also change, resulting in changes in leaf capacitance, leaf resistance and leaf impedance. The unique relationships between WUE and electrophysiological parameters indicate the water deficit of plants. The physiological capacitance of *M. alba* and *B. papyrifera* is negatively correlated with WUE; the higher the physiological capacitance is, the lower the WUE, indicating that as the physiological capacitance increases, the stomata open, stomatal conductance and the transpiration rate increase, the leaf WUE decreases, and the demand for soil moisture is weakened. At the same time, WUE reflects the unique relationship between the physiological impedance and photosynthetic index of the two plants, because the physiological impedance of plants functions to maintain the internal stability of plant cells [42]. The higher the physiological impedance is, the more stomatal closure will reduce transpiration, while WUE will increase. Therefore, the greater the physiological impedance is, the higher the WUE is and the lower the soil water is, and the electrophysiological index can reflect the soil water demand. This confirms the results of the experiment, which showed that the electrophysiological parameters of *M. alba* reflected the water requirement. Furthermore, because the electrophysiological parameters were strongly correlated with the photosynthetic parameters, the photosynthetic parameters could also reflect the water demand of *M. alba*. The water requirements of *B. papyrifera* can also be inferred by the electrophysiological indicators. However, the electrophysiological indexes were not related to the photosynthetic indexes, so the photosynthetic indexes cannot reflect the water demand of plants. The water demand of *B. papyrifera* can be reflected only by the electrophysiological indexes and growth indexes.

Concluded from the above, plant electrophysiological parameters have more advantages in characterizing plant drought resistance. Therefore, electrophysiological parameters can substitute for growth, and photosynthetic parameters to characterize the response of mulberry and paper mulberry to drought.

## 4. Materials and Methods

### 4.1. Plant Growth and Treatment

The experiment was carried out in the greenhouse of the Key Laboratory of Modern Agricultural Equipment and Technology of the Ministry of Education, School of Agricultural Engineering, Jiangsu University (N 32°11′ and E 119°27′). Intact seeds of *Morus alba* L. and *Broussonetia papyrifera* (L.) Vent. were sown and germinated in pots containing equal amounts of peat and perlite on March 8, and irrigated with moderate amounts of water. The pots were kept in trays with drainage holes. When the seedlings reached the 3–5-leaf stage, those with uniform growth were transplanted into pots and cultured. After 2 months of seedling acclimation, water control by the weighing method was started in the evening on May 30. Four treatments were set up for both plant species to control the soil moisture content at 70–75% (CK: the control group, referred to as T0), 55–60% (T1: mild drought), 40–45% (T2: moderate drought), and 25–30% (T3: severe drought). Each drought level consisted of three replicates. Water control was carried out for each treatment every day. The measuring time was once every other week from the beginning of the treatment, and the samples were taken in five times, that is, measurements began on 1 June, after 7d of drought stress for 8 June, 14 d of drought stress for 15 June, 21 d of drought stress for 22 June, 28 d of drought stress for 29 June. When the growth of the plants was basically consistent, the plant growth parameters were measured every two days, the photosynthetic and electrophysiological parameters of the plants were measured every other week for a total of 5 times, taken 3 leaves to calculate the mean value.

### 4.2. Determination of Soil Water Content

The drying method was used to measure the soil relative water content. Soil samples were collected with a small shovel into an aluminum box, and the soil samples were weighed with an analytical balance (0.0001 g precision), to obtain the fresh weight of the soil (m_f_). The samples were dried in a 105 °C oven for 6–8 h to a constant weight. Then, the dry weight of each soil sample (m_d_), expressed by the formula ξ(H_2_O) = (m_f_ − m_d_)/m_d_ × 100%, was determined.

### 4.3. Determination of Growth Parameters

Growth parameters were measured after treatment application, and three data points were recorded each time. The average value was taken as the growth parameter value of the leaves. The measurements taken for growth analysis were plant height, leaf length and leaf width [69]. S-shaped curves are mainly used to describe the natural growth process of animals and plants and are also known as growth curves. The logistic equation with four parameters best described the growth of the plants [48]. The 4-parameter logistic equation is:

(1) $$Y=Y_{0}+\frac{a}{1+{\left(X/X_{0}\right)}^{b}}$$

where *Y*_0_ is the initial logarithmic growth period; a is the upper limit of the growth index of the whole growth process; *X*_0_ is the time it takes to reach 50% of the maximum increase in the logarithmic growth period (days); the growth rate in the half of the logarithmic phase (denoted by GR_50_) is GR_50_ = −*ab*/4*X*_0_; the duration of the logarithmic phase (denoted by DT_log_) is DT_log_ = −4*X*_0_/*b*; and the time from observation to logarithmic growth (denoted by DT_s_) is DT_s_ = *X*_0_ + 2*X*_0_/*b*.

### 4.4. Determination of Leaf Photosynthetic Parameters

The net photosynthetic rate (*P*_N_, µmol(CO_2_)m^−2^s^−1^), stomatal conductance (*g_s_*, mol⋅m^−2^s^−1^), transpiration rate (*Tr*, mmol⋅m^−2^s^−1^), and intercellular CO_2_ concentration (*C*_i_, µmol⋅mol^−1^) were measured with an LI-6400 portable gas exchange measurement system (LI-COR, Lincoln, NE, USA) equipped with a blue/red light source [75,76]. The measurement conditions were as follows: light intensity of 800 μmol·s^−1^, CO_2_ concentration of 400 μmol·mol^−1^, environment temperature of 25 ± 2 °C, the leaf temperature was 30 ± 2 °C, relative humidity of air of 55 ± 5%. These parameters were measured from 9 a.m. to 11 a.m. every seven days under drought treatment. Three leaves were taken from each treatment, and five replicates were taken from each leaf. Water use efficiency (WUE) was calculated according to the equation WUE = *P*_N_/*Tr*, where *P*_N_ is the net photosynthetic rate and *Tr* is the transpiration rate.

### 4.5. Determination of Leaf Electrophysiological Parameters

**Figure 9:**
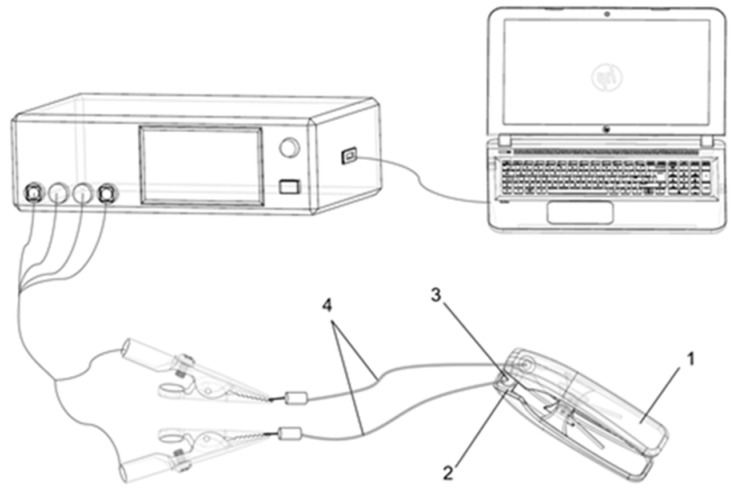
Schematic of the experimental setup. (1) Parallel plate capacitor; (2) foam board; (3) electrode; (4) wire.

Determination of leaf electrophysiological parameters was carried out according to the method described by Xing et al. [58], with some modifications. The instruments (Figure 9) included a LCR tester (Model 3532-50, Hioki, Nagano, Japan), a PC (ThinkPad 1430, lenovo, Beijing, China), homemade parallel plate capacitor (1), foam plate (2), capacitor circular electrode plate (3), and wire with a diameter of 10 mm (4). The parallel plate capacitor was connected to the LCR tester with wire, and the LCR tester was connected to the PC. The leaves on the new branches of the plant were selected, and the leaves were clamped between two parallel electrode plates, avoiding the main leaf vein, while connecting the LCR tester and keep the position of the two electrode plates still. The capacitance (*C*_p_), resistance (R), and impedance (Z) were measured using the LCR tester, the frequency and voltage used were 3 KHz and 1.5 V, respectively. Three to five evenly placed points were selected on the leaves of *M. alba* and *B. papyrifera*, and 10 measurements were taken in each test. That is, each tested leaf yielded 30 to 50 points (the selected parts of each leaf were guaranteed to be the same), and the experimental data were averaged.

### 4.6. Statistical Analysis

Data were analyzed using exploratory data analysis by SPSS software (version 21.0, SPSS Inc., Chicago, IL, USA) and SigmaPlot software (version 10.0, Systat Software Inc., San Jose, CA, USA). The statistical analysis included a one-way analysis of variance (ANOVA), and significant differences between the means were tested using Tukey test at 95% confidence. The data are shown as the means ± SE. Graphs were prepared using Origin Pro. 9.0 (Northampton, MA, USA).

## 5. Conclusions

In both *M. alba* and *B. papyrifera*, plant electrophysiological information can be used to quickly describe the response of plants to soil water deficit. There were obvious correlations between the electrophysiological and photosynthetic indexes of *M. alba*, so these indexes can be used together to represent the response of *M. alba* growth and development to soil water. Except for physiological resistance, the electrophysiological indexes of *B. papyrifera* did not show obvious correlations with the photosynthetic indexes, but in general, the growth indexes of *B. papyrifera* were parallel to the electrophysiological indexes. Therefore, the growth and electrophysiological indexes can be combined to characterize the response of *B. papyrifera* growth and development to soil water. Mild drought had no significant effects on the growth and development of *M. alba* and *B. papyrifera*.

## Figures and Tables

**Table 1:** The soil moisture content experienced by *M. alba* and *B. papyrifera* under different water stress levels.

Plant	Treatment	*ξ*(H_2_O)(%)
*M. alba*	T0	17.70 ± 0.52a
	T1	14.83 ± 0.83b
	T2	10.78 ± 0.45c
	T3	6.90 ± 0.63d
*B. papyrifera*	T0	21.39 ± 0.37a
	T1	14.30 ± 0.72b
	T2	9.89 ± 0.66c
	T3	6.64 ± 0.51d

Note: The means ± SE (n = 5) in the table indicate significant differences in soil moisture content during different water stress phases at *p* ≤ 0.05, according to one-way ANOVA and Tukey-test. *ξ*(H_2_O) is the soil moisture content in %, expressed by the formula *ξ*(H_2_O) = (m_f_ − m_d_)/m_d_ × 100%, where m_f_ (g) and m_d_ (g) are the fresh weight of soil and the dry weight of soil, respectively. T0 is the control group, T1 is the mild drought, T2 is the moderate drought, T3 is the severe drought.

**Table 2:** *M. alba* growth parameters estimated using equations under different water stress levels.

Measurement	Treatment	X_0_	a	GR_50_	DT_log_	DT_s_
Plant height	T0	14.10 ± 0.51ab	91.03 ± 6.70	5.30 ± 0.21b	17.26 ± 1.60	5.47 ± 0.53
	T1	15.95 ± 0.60a	115.25 ± 18.87	5.89 ± 0.31b	19.37 ± 2.27	6.60 ± 0.32
	T2	12.81 ± 0.36b	91.37 ± 8.06	7.01 ± 0.24a	13.07 ± 1.29	6.27 ± 0.50
	T3	13.19 ± 0.29b	86.54 ± 4.40	6.39 ± 0.23ab	13.60 ± 1.00	6.39 ± 0.22
Significant difference			ns		ns	ns
Leaf length	T0	4.33 ± 0.02b	9.86 ± 0.70	2.04 ± 0.29	4.97 ± 0.43	1.85 ± 0.20
	T1	4.87 ± 0.16ab	12.57 ± 1.40	1.88 ± 0.11	6.83 ± 1.07	1.46 ± 0.38
	T2	4.44 ± 0.17b	10.18 ± 0.06	2.09 ± 0.06	4.88 ± 0.13	2.00 ± 0.11
	T3	5.16 ± 0.08a	13.14 ± 1.40	2.51 ± 0.05	5.22 ± 0.45	2.55 ± 0.30
Significant difference			ns	ns	ns	ns
Leaf width	T0	4.40 ± 0.05ab	9.28 ± 0.31	2.22 ± 0.08	4.18 ± 0.06	2.31 ± 0.04a
	T1	4.34 ± 0.25ab	7.45 ± 0.22	1.39 ± 0.15	5.49 ± 0.70	1.59 ± 0.15b
	T2	3.74 ± 0.07b	7.92 ± 0.45	1.63 ± 0.15	4.89 ± 0.23	1.30 ± 0.12b
	T3	4.84 ± 0.35a	8.54 ± 0.97	1.70 ± 0.38	5.33 ± 0.82	2.18 ± 0.06a
Significant difference			ns	ns	ns	

Note: The means ± SE (n = 3) in the table indicate significant differences in *M. alba* growth parameters estimated during different water stress phases at *p* ≤ 0.05, according to one-way ANOVA and Tukey-test. Different lowercase letters in the same column indicate significant differences in the measured factor among the four levels. a is the upper limit of the growth index of the whole growth process; X_0_ is the time it takes to reach 50% of the maximum increase in the logarithmic growth period (days); GR_50_ is the growth rate in the half of the logarithmic phase; DT_log_ is the duration of the logarithmic phase; and DT_s_ is the time from observation to logarithmic growth. ns is no significant differences. T0 is the control group, T1 is the mild drought, T2 is the moderate drought, T3 is the severe drought.

**Table 3:** *B. papyrifera* growth parameters estimated using equations under different water stress levels.

Measurement	Treatment	X_0_	a	GR_50_	DT_log_	DT_s_
Plant height	T0	13.36 ± 0.59	102.54 ± 4.62a	7.29 ± 0.82	14.33 ± 1.32	6.20 ± 0.12
	T1	11.68 ± 0.69	83.94 ± 3.87b	6.45 ± 0.24	13.01 ± 0.13	5.18 ± 0.76
	T2	12.13 ± 0.02	87.14 ± 2.25ab	5.91 ± 0.65	15.16 ± 1.87	4.55 ± 0.94
	T3	13.37 ± 0.44	94.75 ± 2.96ab	5.97 ± 0.39	15.98 ± 1.02	5.38 ± 0.83
Significant difference		ns		ns	ns	ns
Leaf length	T0	4.56 ± 0.10	133.05 ± 3.82a	27.33 ± 1.02	4.87 ± 0.10	2.12 ± 0.08
	T1	4.67 ± 0.20	133.48 ± 9.79a	27.62 ± 2.50	4.85 ± 0.21	2.04 ± 0.23
	T2	3.78 ± 0.14	97.58 ± 2.10b	25.53 ± 0.25	3.82 ± 0.08	1.87 ± 0.09
	T3	3.86 ± 0.26	76.25 ± 6.47b	19.72 ± 2.12	4.17 ± 0.75	1.77 ± 0.12
Significant difference		ns		ns	ns	ns
Leaf width	T0	4.94 ± 0.23a	170.30 ± 8.64a	32.79 ± 0.74	5.20 ± 0.23	2.34 ± 0.17a
	T1	4.93 ± 0.10a	157.05 ± 2.04a	29.70 ± 0.65	5.30 ± 0.18	2.28 ± 0.02ab
	T2	3.95 ± 0.04b	112.15 ± 4.77b	26.03 ± 2.94	4.37 ± 0.28	1.77 ± 0.18b
	T3	4.36 ± 0.18ab	120.03 ± 1.51b	25.24 ± 1.36	4.78 ± 0.28	1.97 ± 0.04ab
Significant difference				ns	ns	

Note: The means ± SE (n = 3) in the table indicate significant differences in *B. papyrifera* growth parameters estimated during different water stress phases at *p* ≤ 0.05, according to one-way ANOVA and Tukey-test. Different lowercase letters in the same column indicate significant differences in the measured factor among the four levels. a is the upper limit of the growth index of the whole growth process; X_0_ is the time it takes to reach 50% of the maximum increase in the logarithmic growth period (days); GR_50_ is the growth rate in the half of the logarithmic phase; DT_log_ is the duration of the logarithmic phase; and DT_s_ is the time from observation to logarithmic growth. ns is no significant differences. T0 is the control group, T1 is the mild drought, T2 is the moderate drought, T3 is the severe drought.

**Table 4:** Effects of different water stress levels on the plant height, leaf length, leaf width and leaf area of *M. alba*.

Treatment	Plant H. (mm) GR_50_ × DT_log_	L_leaf_ (mm) GR_50_ × DT_log_	W_leaf_ (mm) GR_50_ × DT_log_	A_leaf_ (×10^2^ mm^2^) L_leaf_ × W_leaf_
T0	91.05 ± 6.67	9.88 ± 0.70	9.28 ± 0.31	91.44 ± 5.21
T1	115.22 ± 18.86	12.59 ± 1.40	7.43 ± 0.22	92.94 ± 8.14
T2	91.38 ± 8.04	10.16 ± 0.08	7.92 ± 0.44	80.49 ± 4.69
T3	86.56 ± 4.42	13.14 ± 1.40	8.55 ± 0.10	114.40 ± 23.74
Significant difference	ns	ns	ns	ns

Note: The means ± SE (n = 3) in the table indicate significant differences in *M. alba* growth parameters estimated during different water stress phases at *p* ≤ 0.05, according to one-way ANOVA and Tukey-test. Different lowercase letters in the same column indicate significant differences in the measured factor among the four levels. Plant H.: plant height. L_leaf_: leaf length. W_leaf:_ leaf width. A_leaf_: leaf area. GR_50_ × DT_log_ is the total growth of the logarithmic phase. ns is no significant differences. T0 is the control group, T1 is the mild drought, T2 is the moderate drought, T3 is the severe drought.

**Table 5:** Effects of different water stress levels on the plant height, leaf length, leaf width and leaf area of *B. papyrifera*.

Treatment	Plant H. (mm) GR_50_ × DT_log_	L_leaf_ (mm) GR_50_ × DT_log_	W_leaf_ (mm) GR_50_ × DT_log_	A_leaf_ (×10^2^ mm^2^) L_leaf_ × W_leaf_
T0	102.51 ± 4.59a	133.04 ± 3.83a	170.42 ± 8.66a	226.31 ± 9.25a
T1	83.95 ± 3.90b	133.27 ± 9.79a	157.07 ± 2.05a	208.94 ± 12.57a
T2	87.10 ± 2.27ab	97.61 ± 2.08b	112.09 ± 4.80b	109.61 ± 7.09b
T3	94.76 ± 3.00ab	76.23 ± 6.47b	120.01 ± 1.55b	91.28 ± 6.55b

Note: The means ± SE (n = 3) in the table indicate significant differences in *B. papyrifera* growth parameters estimated during different water stress phases at *p* ≤ 0.05, according to one-way ANOVA and Tukey-test. Different lowercase letters in the same column indicate significant differences in the measured factor among the four levels. Plant H.: plant height. L_leaf_: leaf length. W_leaf:_ leaf width. A_leaf_: leaf area. GR_50_ × DT_log_ is the total growth of the logarithmic phase. T0 is the control group, T1 is the mild drought, T2 is the moderate drought, T3 is the severe drought.

**Table 6:** Pearson correlation coefficients between different physiological parameters of *M. alba* (*n* = 60).

	*g_s_*	*C*_i_	*Tr*	WUE	*C*_p_	R	Z
*P*_N_	0.888 **	0.685 **	0.850 **	−0.319 *	0.380 **	−0.425 **	−0.192
*g_s_*		0.834 **	0.820 **	−0.413 **	0.546 **	−0.532 **	−0.236
*C*_i_			0.573 **	−0.280 *	0.480 **	−0.482 **	−0.088
*Tr*				−0.709 **	0.544 **	−0.464 **	−0.311 *
WUE					−0.585 **	0.395 **	0.410 **
*C*_p_						−0.892 **	−0.545 **
R							0.600 **

Note: ** Correlation is significant at the 0.01 level. Two-tailed significance was used. * Correlation is significant at the 0.05 level. Two-tailed significance was used. *P*_N_: the net photosynthetic rate. *g_s_*: the stomatal conductance. *C*_i_: the intercellular CO_2_ concentration. *Tr*: the transpiration rate. WUE: the water use efficiency. *C*_p_: the physiological capacitance. R: the physiological resistance. Z: the physiological impedance.

**Table 7:** Pearson correlation coefficients among different physiological parameters of *B. papyrifera* (*n* = 60).

	*g_s_*	*C*_i_	*Tr*	WUE	*C*_p_	R	Z
*P*_N_	0.861 **	0.535 **	0.719 **	−0.011	0.031	−0.236	−0.043
*g_s_*		0.725 **	0.753 **	−0.153	0.148	−0.289 *	−0.144
*C*_i_			0.356 **	0.114	0.205	−0.369 **	−0.057
*Tr*				−0.684 **	0.221	−0.212	−0.294 *
WUE					−0.281 *	0.039	0.445 **
*C*_p_						−0.548 **	−0.712 **
R							0.471 **

Note: ** Correlation is significant at the 0.01 level. Two-tailed significance was used. * Correlation is significant at the 0.05 level. Two-tailed significance was used. *P*_N_: the net photosynthetic rate. *g_s_*: the stomatal conductance. *C*_i_: the intercellular CO_2_ concentration. *Tr*: the transpiration rate. WUE: the water use efficiency. *C*_p_: the physiological capacitance. R: the physiological resistance. Z: the physiological impedance.

## Data Availability

The datasets during or analyzed during the current study available from the corresponding author on reasonable request.

## Appendix A

**Table A1:** *M. alba* growth parameters estimated using equation under different water stresses.

Measurement	Treatment	X_0_	a	GR_50_	DT_log_	DT_s_	Equation and R^2^
Plant height	T0	14.83	102.95	5.03	20.46	4.60	Y = 33.52 + $\frac{102.95}{1+{\left(x/14.83\right)}^{-2.90}}$, R^2^ = 0.9990 (*n* = 29, *p* < 0.0001)
		13.11	79.75	5.16	15.47	5.38	Y = 36.63 + $\frac{79.75}{1+{\left(x/13.11\right)}^{-3.39}}$, R^2^ = 0.9982 (*n* = 29, *p* < 0.0001)
		14.35	90.39	5.70	15.86	6.42	Y = 32.06 + $\frac{90.39}{1+{\left(x/14.35\right)}^{-3.62}}$, R^2^ = 0.9978 (*n* = 29, *p* < 0.0001)
	T1	16.34	115.21	5.69	20.24	6.22	Y = 37.96 + $\frac{115.21}{1+{\left(x/16.34\right)}^{-3.23}}$, R^2^ = 0.9987 (*n* = 29, *p* < 0.0001)
		16.75	147.95	6.49	22.79	6.36	Y = 32.36 + $\frac{147.95}{1+{\left(x/16.75\right)}^{-2.94}}$, R^2^ = 0.9990 (*n* = 29, *p* < 0.0001)
		14.77	82.59	5.48	15.07	7.23	Y = 35.43 + $\frac{82.59}{1+{\left(x/14.77\right)}^{-3.92}}$, R^2^ = 0.9975 (*n* = 29, *p* < 0.0001)
	T2	13.22	107.00	6.84	15.64	5.40	Y = 34.59 + $\frac{107.00}{1+{\left(x/13.22\right)}^{-3.38}}$, R^2^ = 0.9985 (*n* = 29, *p* < 0.0001)
		13.11	80.11	6.71	11.95	7.14	Y = 34.20 + $\frac{80.11}{1+{\left(x/13.11\right)}^{-4.39}}$, R^2^ = 0.9947 (*n* = 29, *p* < 0.0001)
		12.09	87.00	7.48	11.63	6.28	Y = 38.16 + $\frac{87.00}{1+{\left(x/12.09\right)}^{-4.16}}$, R^2^ = 0.9975 (*n* = 29, *p* < 0.0001)
	T3	13.74	95.28	6.21	15.35	6.06	Y = 32.83 + $\frac{95.28}{1+{\left(x/13.74\right)}^{-3.58}}$, R^2^ = 0.9989 (*n* = 29, *p* < 0.0001)
		12.73	81.22	6.84	11.87	6.80	Y = 32.96 + $\frac{81.22}{1+{\left(x/12.73\right)}^{-4.29}}$, R^2^ = 0.9967 (*n* = 29, *p* < 0.0001)
		13.11	83.13	6.12	13.59	6.32	Y = 34.25 + $\frac{83.13}{1+{\left(x/13.11\right)}^{-3.86}}$, R^2^ = 0.9983 (*n* = 29, *p* < 0.0001)
Leaf length	T0	4.37	8.77	1.51	5.83	1.46	Y = 40.18 + $\frac{8.77}{1+{\left(x/4.37\right)}^{-3.00}}$, R^2^ = 0.9996 (*n* = 10, *p* < 0.0001)
		4.30	11.17	2.51	4.46	2.07	Y = 42.21 + $\frac{11.17}{1+{\left(x/4.30\right)}^{-3.86}}$, R^2^ = 0.9990 (*n* = 10, *p* < 0.0001)
		4.32	9.65	2.09	4.61	2.02	Y = 46.21 + $\frac{9.65}{1+{\left(x/4.32\right)}^{-3.75}}$, R^2^ = 0.9989 (*n* = 10, *p* < 0.0001)
	T1	5.03	14.00	1.72	8.15	0.96	Y = 39.92 + $\frac{14.00}{1+{\left(x/5.03\right)}^{-2.47}}$, R^2^ = 0.9993 (*n* = 10, *p* < 0.0001)
		4.56	9.78	2.08	4.70	2.21	Y = 38.83 + $\frac{9.78}{1+{\left(x/4.56\right)}^{-3.88}}$, R^2^ = 0.9992 (*n* = 10, *p* < 0.0001)
		5.02	13.94	1.83	7.63	1.20	Y = 46.27 + $\frac{13.94}{1+{\left(x/5.02\right)}^{-2.63}}$, R^2^ = 0.9992 (*n* = 10, *p* < 0.0001)
	T2	4.11	10.14	2.16	4.68	1.77	Y = 42.54 + $\frac{10.14}{1+{\left(x/4.11\right)}^{-3.51}}$, R^2^ = 0.9982 (*n* = 10, *p* < 0.0001)
		4.66	10.09	1.96	5.13	2.09	Y = 34.67 + $\frac{10.09}{1+{\left(x/4.66\right)}^{-3.63}}$, R^2^ = 0.9994 (*n* = 10, *p* < 0.0001)
		4.54	10.30	2.14	4.82	2.13	Y = 41.68 + $\frac{10.30}{1+{\left(x/4.54\right)}^{-3.77}}$, R^2^ = 0.9991 (*n* = 10, *p* < 0.0001)
	T3	5.30	11.55	2.48	4.66	2.97	Y = 37.71 + $\frac{11.55}{1+{\left(x/5.30\right)}^{-4.55}}$, R^2^ = 0.9979 (*n* = 10, *p* < 0.0001)
		5.15	11.94	2.44	4.89	2.70	Y = 32.07 + $\frac{11.94}{1+{\left(x/5.15\right)}^{-4.21}}$, R^2^ = 0.9971 (*n* = 10, *p* < 0.0001)
		5.03	15.93	2.61	6.10	1.98	Y = 28.53 + $\frac{15.93}{1+{\left(x/5.03\right)}^{-3.30}}$, R^2^ = 0.9996 (*n* = 10, *p* < 0.0001)
Leaf width	T0	4.49	9.88	2.34	4.23	2.38	Y = 32.77 + $\frac{9.88}{1+{\left(x/4.49\right)}^{-4.25}}$, R^2^ = 0.9993 (*n* = 10, *p* < 0.0001)
		4.32	9.10	2.24	4.06	2.29	Y = 35.42 + $\frac{9.10}{1+{\left(x/4.32\right)}^{-4.26}}$, R^2^ = 0.9987 (*n* = 10, *p* < 0.0001)
		4.38	8.87	2.08	4.26	2.25	Y = 37.79 + $\frac{8.87}{1+{\left(x/4.38\right)}^{-4.11}}$, R^2^ = 0.9983 (*n* = 10, *p* < 0.0001)
	T1	4.00	7.13	1.68	4.24	1.88	Y = 34.27 + $\frac{7.13}{1+{\left(x/4.00\right)}^{-3.77}}$, R^2^ = 0.9977 (*n* = 10, *p* < 0.0001)
		4.82	7.87	1.18	6.65	1.50	Y = 31.72 + $\frac{7.87}{1+{\left(x/4.82\right)}^{-2.90}},$ R^2^ = 0.9977 (*n* = 10, *p* < 0.0001)
		4.19	7.34	1.31	5.59	1.40	Y = 39.74 + $\frac{7.34}{1+{\left(x/4.19\right)}^{-3.00}}$, R^2^ = 0.9991 (*n* = 10, *p* < 0.0001)
	T2	3.65	7.02	1.37	5.14	1.08	Y = 30.05 + $\frac{7.02}{1+{\left(x/3.65\right)}^{-2.84}}$, R^2^ = 0.9988 (*n* = 10, *p* < 0.0001)
		3.70	8.38	1.89	4.43	1.48	Y = 29.71 + $\frac{8.38}{1+{\left(x/3.70\right)}^{-3.34}}$, R^2^ = 0.9964 (*n* = 10, *p* < 0.0001)
		3.88	8.37	1.64	5.09	1.34	Y = 35.93 + $\frac{8.37}{1+{\left(x/3.88\right)}^{-3.05}}$, R^2^ = 0.9962 (*n* = 10, *p* < 0.0001)
	T3	4.74	6.68	1.32	5.07	2.21	Y = 29.81 + $\frac{6.68}{1+{\left(x/4.74\right)}^{-3.74}}$, R^2^ = 0.9977 (*n* = 10, *p* < 0.0001)
		5.49	8.99	1.31	6.86	2.06	Y = 23.96 + $\frac{8.99}{1+{\left(x/5.49\right)}^{-3.20}}$, R^2^ = 0.9986 (*n* = 10, *p* < 0.0001)
		4.29	9.96	2.46	4.05	2.27	Y = 21.78 + $\frac{9.96}{1+{\left(x/4.29\right)}^{-4.24}}$, R^2^ = 0.9989 (*n* = 10, *p* < 0.0001)

**Table A2:** *B. papyrifera* growth parameters estimated using equation under different water stresses.

Measurement	Treatment	X_0_	a	GR_50_	DT_log_	DT_s_	Equation and R^2^
Plant height	T0	14.40	108.21	6.59	16.41	6.19	Y = 22.73 + $\frac{108.21}{1+{\left(x/14.40\right)}^{-3.51}}$, R^2^ = 0.9992 (*n* = 29, *p* < 0.0001)
		13.33	93.38	6.36	14.69	5.99	Y = 30.27 + $\frac{93.38}{1+{\left(x/13.33\right)}^{-3.63}}$, R^2^ = 0.9990 (*n* = 29, *p* < 0.0001)
		12.36	106.03	8.92	11.88	6.42	Y = 30.19 + $\frac{106.03}{1+{\left(x/12.36\right)}^{-4.16}}$, R^2^ = 0.9978 (*n* = 29, *p* < 0.0001)
	T1	12.79	76.40	5.97	12.79	6.4	Y = 34.38 + $\frac{76.40}{1+{\left(x/12.79\right)}^{-4.00}}$, R^2^ = 0.9988 (*n* = 29, *p* < 0.0001)
		11.84	86.20	6.64	12.98	5.35	Y = 26.31 + $\frac{86.20}{1+{\left(x/11.84\right)}^{-3.65}}$, R^2^ = 0.9989 (*n* = 29, *p* < 0.0001)
		10.40	89.23	6.74	13.25	3.78	Y = 27.39 + $\frac{89.23}{1+{\left(x/10.40\right)}^{-3.14}}$, R^2^ = 0.9978 (*n* = 29, *p* < 0.0001)
	T2	12.16	91.05	6.01	15.15	4.58	Y = 34.12 + $\frac{91.05}{1+{\left(x/12.16\right)}^{-3.21}}$, R^2^ = 0.9955 (*n* = 29, *p* < 0.0001)
		12.10	87.11	4.73	18.40	2.90	Y = 28.55 + $\frac{87.11}{1+{\left(x/12.10\right)}^{-2.63}}$, R^2^ = 0.9974 (*n* = 29, *p* < 0.0001)
		12.13	83.26	6.98	11.92	6.17	Y = 30.68 + $\frac{83.26}{1+{\left(x/12.13\right)}^{-4.07}}$, R^2^ = 0.9963 (*n* = 29, *p* < 0.0001)
	T3	13.94	93.87	6.72	13.97	6.95	Y = 29.72 + $\frac{93.87}{1+{\left(x/13.94\right)}^{-3.99}}$, R^2^ = 0.9976 (*n* = 29, *p* < 0.0001)
		12.51	90.11	5.38	16.74	4.14	Y = 36.09 + $\frac{90.11}{1+{\left(x/12.51\right)}^{-2.99}}$, R^2^ = 0.9967 (*n* = 29, *p* < 0.0001)
		13.66	100.26	5.82	17.24	5.04	Y = 38.14 + $\frac{100.26}{1+{\left(x/13.66\right)}^{-3.17}}$, R^2^ = 0.9992 (*n* = 29, *p* < 0.0001)
Leaf length	T0	4.75	138.25	27.65	5.00	2.25	Y = 34.03 + $\frac{138.25}{1+{\left(x/4.75\right)}^{-3.80}}$, R^2^ = 0.9958 (*n* = 10, *p* < 0.0001)
		4.46	125.61	25.42	4.94	1.99	Y = 40.23 + $\frac{125.61}{1+{\left(x/4.46\right)}^{-3.61}}$, R^2^ = 0.9979 (*n* = 10, *p* < 0.0001)
		4.47	135.30	28.91	4.68	2.13	Y = 38.01 + $\frac{135.30}{1+{\left(x/4.47\right)}^{-3.82}}$, R^2^ = 0.9967 (*n* = 10, *p* < 0.0001)
	T1	4.86	152.59	31.63	4.82	2.45	Y = 40.01 + $\frac{152.59}{1+{\left(x/4.86\right)}^{-4.03}}$, R^2^ = 0.9944 (*n* = 10, *p* < 0.0001)
		4.26	127.61	28.18	4.51	2.01	Y = 31.27 + $\frac{127.61}{1+{\left(x/4.26\right)}^{-3.78}}$, R^2^ = 0.9967 (*n* = 10, *p* < 0.0001)
		4.28	120.25	23.04	5.22	1.67	Y = 37.75 + $\frac{120.25}{1+{\left(x/4.28\right)}^{-3.28}}$, R^2^ = 0.9977 (*n* = 10, *p* < 0.0001)
	T2	4.01	101.69	25.74	3.95	2.03	Y = 36.55 + $\frac{101.69}{1+{\left(x/4.01\right)}^{-4.06}}$, R^2^ = 0.9958 (*n* = 10, *p* < 0.0001)
		3.54	94.74	25.83	3.67	1.71	Y = 37.08 + $\frac{94.74}{1+{\left(x/3.54\right)}^{-3.86}}$, R^2^ = 0.9988 (*n* = 10, *p* < 0.0001)
		3.80	96.32	25.03	3.85	1.88	Y = 40.85 + $\frac{96.32}{1+{\left(x/3.80\right)}^{-3.95}}$, R^2^ = 0.9962 (*n* = 10, *p* < 0.0001)
	T3	4.36	66.06	11.67	5.66	1.53	Y = 37.26 + $\frac{66.06}{1+{\left(x/4.36\right)}^{-3.08}}$, R^2^ = 0.9990 (*n* = 10, *p* < 0.0001)
		3.70	88.25	25.28	3.49	1.95	Y = 32.06 + $\frac{88.25}{1+{\left(x/3.70\right)}^{-4.24}}$, R^2^ = 0.9950 (*n* = 10, *p* < 0.0001)
		3.51	74.43	22.21	3.35	1.83	Y = 42.05 + $\frac{74.43}{1+{\left(x/3.51\right)}^{-4.19}}$, R^2^ = 0.9970 (*n* = 10, *p* < 0.0001)
Leaf width	T0	4.50	153.04	31.97	4.79	2.11	Y = 29.99 + $\frac{153.04}{1+{\left(x/4.50\right)}^{-3.76}}$, R^2^ = 0.9993 (*n* = 10, *p* < 0.0001)
		5.26	178.06	34.27	5.20	2.66	Y = 38.32 + $\frac{178.06}{1+{\left(x/5.26\right)}^{-4.05}}$, R^2^ = 0.9936 (*n* = 10, *p* < 0.0001)
		5.05	179.79	32.13	5.60	2.25	Y = 39.96 + $\frac{179.79}{1+{\left(x/5.05\right)}^{-3.61}}$, R^2^ = 0.9952 (*n* = 10, *p* < 0.0001)
	T1	4.76	153.32	30.60	5.01	2.25	Y = 35.51 + $\frac{153.32}{1+{\left(x/4.76\right)}^{-3.80}}$, R^2^ = 0.9957 (*n* = 10, *p* < 0.0001)
		4.95	157.49	30.07	5.24	2.33	Y = 40.42 + $\frac{157.49}{1+{\left(x/4.95\right)}^{-3.78}}$, R^2^ = 0.9923 (*n* = 10, *p* < 0.0001)
		5.09	160.34	28.43	5.64	2.27	Y = 43.07 + $\frac{160.34}{1+{\left(x/5.09\right)}^{-3.61}}$, R^2^ = 0.9985 (*n* = 10, *p* < 0.0001)
	T2	4.03	121.67	31.85	3.82	2.12	Y = 47.11 + $\frac{121.67}{1+{\left(x/4.03\right)}^{-4.22}}$, R^2^ = 0.9963 (*n* = 10, *p* < 0.0001)
		3.95	106.78	22.37	4.77	1.56	Y = 46.70 + $\frac{106.78}{1+{\left(x/3.95\right)}^{-3.31}}$, R^2^ = 0.9986 (*n* = 10, *p* < 0.0001)
		3.88	108.00	23.87	4.52	1.62	Y = 37.27 + $\frac{108.00}{1+{\left(x/3.88\right)}^{-3.43}}$, R^2^ = 0.9987 (*n* = 10, *p* < 0.0001)
	T3	4.68	122.57	23.18	5.29	2.04	Y = 29.20 + $\frac{122.57}{1+{\left(x/4.68\right)}^{-3.54}}$, R^2^ = 0.9967 (*n* = 10, *p* < 0.0001)
		4.34	117.35	24.74	4.74	1.97	Y = 51.78 + $\frac{117.35}{1+{\left(x/4.34\right)}^{-3.66}}$, R^2^ = 0.9971 (*n* = 10, *p* < 0.0001)
		4.05	120.16	27.81	4.32	1.89	Y = 32.24 + $\frac{120.16}{1+{\left(x/4.05\right)}^{-3.75}}$, R^2^ = 0.9969 (*n* = 10, *p* < 0.0001)
